# Alerts for community pharmacist-provided medication therapy management: recommendations from a heuristic evaluation

**DOI:** 10.1186/s12911-019-0866-0

**Published:** 2019-07-16

**Authors:** Margie E. Snyder, Heather Jaynes, Stephanie A. Gernant, Julie DiIulio, Laura G. Militello, William R. Doucette, Omolola A. Adeoye, Alissa L. Russ

**Affiliations:** 1 Department of Pharmacy Practice, Purdue University College of Pharmacy, 640 Eskenazi Ave, Indianapolis, IN 46220 USA; 2 Department of Pharmacy Practice, University of Connecticut School of Pharmacy, Storrs, CT USA; 3 Applied Decision Science, LLC, Cincinnati, OH USA; 4 Division of Health Services Research, Department of Pharmacy Practice and Science, University of Iowa College of Pharmacy, Iowa City, IA USA; 5 Regenstrief Institute, Inc, Indianapolis, IN USA

**Keywords:** Decision support systems, clinical, Ergonomics, Community pharmacy services, Medication therapy management

## Abstract

**Background:**

Medication therapy management (MTM) is a service, most commonly provided by pharmacists, intended to identify and resolve medication therapy problems (MTPs) to enhance patient care. MTM is typically documented by the community pharmacist in an MTM vendor’s web-based platform. These platforms often include integrated alerts to assist the pharmacist with assessing MTPs. In order to maximize the usability and usefulness of alerts to the end users (e.g., community pharmacists), MTM alert design should follow principles from human factors science. Therefore, the objectives of this study were to 1) evaluate the extent to which alerts for community pharmacist-delivered MTM align with established human factors principles, and 2) identify areas of opportunity and recommendations to improve MTM alert design.

**Methods:**

Five categories of MTM alerts submitted by community pharmacists were evaluated: 1) *indication,* 2) *effectiveness*; 3) *safety*; 4) *adherence*; and 5) *cost-containment*. This heuristic evaluation was guided by the *Instrument for Evaluating Human-Factors Principles in Medication-Related Decision Support Alerts* (I-MeDeSA) which we adapted and contained 32 heuristics. For each MTM alert, four analysts’ individual ratings were summed and a mean score on the modified I-MeDeSA computed. For each heuristic, we also computed the percent of analyst ratings indicating alignment with the heuristic. We did this for all alerts evaluated to produce an “overall” summary of analysts’ ratings for a given heuristic, and we also computed this separately for each alert category. Our results focus on heuristics where ≤50% of analysts’ ratings indicated the alerts aligned with the heuristic.

**Results:**

I-MeDeSA scores across the five alert categories were similar. Heuristics pertaining to visibility and color were generally met. Opportunities for improvement across all MTM alert categories pertained to the principles of alert prioritization; text-based information; alarm philosophy; and corrective actions.

**Conclusions:**

MTM alerts have several opportunities for improvement related to human factors principles, resulting in MTM alert design recommendations. Enhancements to MTM alert design may increase the effectiveness of MTM delivery by community pharmacists and result in improved patient outcomes.

**Electronic supplementary material:**

The online version of this article (10.1186/s12911-019-0866-0) contains supplementary material, which is available to authorized users.

## Background

There are many studies of electronic health record (EHR) medication alerts in hospital systems and physician office settings, but fewer studies have been conducted on medication-related alerts in community pharmacies with pharmacists as end users [1–7]. This area of research is needed given the complexity of medication use in ambulatory and community pharmacy settings. In the Medicare patient population, adverse drug events (ADEs) costs in the ambulatory setting from preventable medication therapy problems (MTPs) are estimated at $887 million annually [8]. Medication therapy management (MTM) is a service, most commonly provided by pharmacists, intended to identify and resolve MTPs to enhance patient care [9]. MTM consists of five key steps: 1) complete a targeted or comprehensive medication review (CMR) to identify MTPs, 2) provide a patient-specific personal medication list, 3) generate and provide a medication-related action plan, 4) intervention/referral, and 5) clinical documentation and follow-up [10].

To promote medication adherence and reduce ADEs among patients meeting specific criteria (e.g., chronically ill, using multiple medications), Medicare Part D plans must offer MTM, which includes an annual CMR. During the CMR, a pharmacist meets with a patient and/or caregiver to review all of the patient’s medications [11]. Part D plans vary in their approach to meeting this requirement with some choosing to have pharmacists employed by the plan provide MTM telephonically while others contract with MTM vendors to provide MTM services through networks of community pharmacists [12]. As of 2018, more than 60% of Part D plans utilize MTM vendor community pharmacy networks for at least some of their MTM-eligible patients [12].

MTM outcomes for patients, such as medication adherence and hospitalizations, have been inconsistent [13, 14]. Some of this variability may be attributed to alert design and functionality. MTM is typically documented by the community pharmacist in an MTM vendor’s web-based platform. These platforms often include integrated alerts to assist the pharmacist with assessing MTPs. For example, an MTM alert could prompt the pharmacist to speak with the patient during the CMR about possible medication non-adherence. Although alert design in the context of MTM has not been well-studied, possible limitations with alerts for MTM and considerations for alert design have been identified by our research group in prior qualitative work [15]. For example, this research found that some MTM alerts (i.e., for assessing medication non-adherence) often falsely identified MTPs [15]. Improving the design of MTM alerts could be a strategy to improve patient outcomes.

In order to maximize the usability and usefulness of alerts to the end users (e.g., community pharmacists), MTM alert design should follow principles from human factors science [16]. The goal of human factors science is to “promote efficiency, safety, and effectiveness by improving the design of technologies, processes, and work systems” so that they support the physical and cognitive capabilities and limitations of humans [16]. Extensive human factors research has been conducted on alert design in other domains including nuclear power [17], aviation [18], unmanned aerial systems [19], air traffic control [20], and hospital/physician office EHR-based medication safety [21]; our intent was to extend this work to MTM alert design.

## Objectives

The study objectives were to 1) evaluate the extent to which alerts for community pharmacist-delivered MTM align with established human factors principles and 2) identify areas of opportunity and recommendations to improve MTM alert design for use in community pharmacies.

## Methods

### Conceptual frameworks

This study was informed by the Systems Engineering Initiative for Patient Safety (SEIPS) v. 2.0 [22]. As described in SEIPS 2.0, six work system components influence work processes, which thereby influence outcomes [22]. One component is “tools and technology,” such as alerts for MTM. In addition, we used the MTP taxonomy described by Cipolle et al. [23] to organize our findings. According to this taxonomy, MTPs can be categorized as related to a medication’s: 1) *indication* (e.g., to assess whether a medication is medically indicated for the patient), 2) *effectiveness* (e.g., a higher dose of medication is recommended), 3) *safety* (e.g., a drug-drug interaction is present that increases the risk of toxicity for the patient), or 4) *medication adherence* (e.g., the patient has difficulty remembering to take their medication.) [23] For this study, we added a MTP category: 5) payer-driven alerts for *cost-containment* purposes (e.g., the alert recommends a brand to generic switch) to align with alerts that are available in MTM vendor platforms. Thus, as described below, we used these five categories to examine how the extent of alert alignment with human factors principles varies by category of MTP targeted by the MTM alert.

### Setting, participants, and MTM alert systems

MTM vendors contract with pharmacies and payers on MTM program delivery. These vendors have developed web-based commercial documentation platforms in which pharmacists document MTM services. These platforms often include integrated alerts to assist the pharmacist in the assessment of MTPs. This study was conducted in collaboration with regional pharmacist/pharmacy practice-based research networks located in two different states: the Medication Safety Research Network of Indiana (Rx-SafeNet) and the Minnesota Pharmacy Practice-based Research Network (MPPBRN) [24, 25]. Community pharmacists from these networks were eligible to participate if they reported that they routinely provided CMRs for at least one of two national MTM vendors that require documentation in a web-based platform with integrated alerts. Eligibility criteria focused on these particular MTM vendors because the researchers were aware of their long-standing use by pharmacists in the collaborating networks. All MTM vendors with alerts evaluated as part of this study have been contracting on MTM program delivery for more than 10 years.

### Study Design & Modification of I-MeDeSA

A heuristic evaluation is “one method for measuring usability … through which experts identify potential usability problems by comparing designs against established principles (i.e., heuristics).” [26] We followed established techniques to conduct a heuristic evaluation of MTM alerts [27–30]. A heuristic evaluation was chosen to understand how well MTM alerts align with established human factors principles and to maximize our ability to identify opportunities to enhance MTM alert design. The heuristic evaluation was guided by a modified version of the *Instrument for Evaluating Human-Factors Principles in Medication-Related Decision Support Alerts* (I-MeDeSA) [31]. The original I-MeDeSA contains 26 heuristics pertaining to human factors principles and was designed to evaluate drug-drug interaction alerts associated with EHRs [21, 31, 32]. Because the original I-MeDeSA focused solely on drug-drug interactions, we modified it to encompass the broader alerts generated during MTM. Our modified version of the I-MeDeSA removed one heuristic and added 7 additional heuristics for a total of 32 heuristics pertaining to eight human factors principles: *alarm philosophy*, *placement*, *visibility*, *prioritization*, *color*, *text-based information*, *proximity of task components being displayed*, and *corrective actions* (Additional file 1). The additional heuristics incorporate usability design heuristics and human factors recommendations related to warning design [33]. The scoring procedures for the I-MeDeSA were also modified to enable indication of heuristics that were “unable to be assessed” from screenshots, to avoid forcing analysts into an inaccurate response and provide insights on areas for future research. Prior to data analysis, the modified I-MeDeSA was piloted by two investigators (a research pharmacist and a human factors professional) with two rounds of review with example MTM alert screenshots.

### Data collection: screenshots of MTM alerts

Following recruitment and training, community pharmacists submitted screenshots of MTM alerts that were generated by the MTM vendor platform as part of community pharmacists’ routine provision of CMRs. Pharmacists used HyperSnap software (Hyperionics, vs. 7 and 8, Boswell, PA) [34] to take screen shots associated with MTM alerts. Specifically, pharmacists were instructed to take screenshots representing their routine workflow for opening and responding to MTM alerts, including the initial screen from which the alert is accessed to the final screen where the alert is resolved or requires later action. They were instructed to redact any protected health information. Pharmacists were asked to submit alert screen shots for five MTP alert categories: 1) *indication,* 2) *effectiveness*; 3) *safety*; 4) *adherence*; and 5) payer-driven alerts for *cost-containment* purposes [23]. Pharmacists submitted screenshots to our team using REDCap, a secure, web-based application developed to support the collection of research data [35]. We asked pharmacists to start by submitting alerts for three patients, preferably with MTM alerts representing varying categories. Submitted MTM screenshots were first reviewed by two investigators (a research pharmacist and nurse.) The purpose of this review was to 1) ensure completeness of the submission (e.g., screenshots for each step in the pharmacist’s workflow) and request clarifying information from pharmacists when needed, and 2) to purposefully sample 1–2 alerts per category per vendor, with a goal of collecting a total of 24 alerts for our evaluation. As data collection proceeded, we provided guidance to pharmacists on categories of alerts that were still needed for data collection goals.

### Data analysis: heuristic evaluation

Prior to analysis, a second pharmacist on the research team verified the categorization of MTM alerts made during the initial review. Four analysts conducted the heuristic evaluation. The analyst team was comprised of three human factors professionals and one pharmacist/MTM domain expert [27, 30]. This mix of analysts is supported by literature on heuristic evaluation, which demonstrates that at least 3 expert reviewers are needed, 3–5 reviewers can identify up to 75% of usability problems, and including domain experts (i.e., MTM, community pharmacy) can further strengthen the rigor and quality of results [27, 30]. Analysts completed a total of 3 h of training which included further pilot testing of the modified I-MeDeSA and minor revisions of the instrument to ensure clarity of heuristics for all analysts. This level of training aligns with recommendations from the literature [30].

Each analyst independently rated each alert on the modified I-MeDeSA. To maintain the same sentiment as scoring for the original I-MeDeSA (i.e., alerts scored a “1” for each heuristic met) but to enable investigators to distinguish between definite alignment and uncertainty, heuristics were scored as “0” when the alert “did not align” with the heuristic, “1” when the analyst was “unable to assess” whether the alert aligned with the heuristic (e.g., if the alert was appropriately timed but no specific timing information was available), and “2” when the “alert aligned” with the heuristic so that increasing scores reflected increased certainty in alert alignment. Therefore, for each evaluated MTM alert, the total possible scores on the modified I-MeDeSA ranged from 0 to 64 (32 heuristics each given a score of 2 would indicate alignment across all heuristics.) Analysts were required to enter explanatory comments when indicating “unable to assess” a heuristic or “not aligned” with the heuristic.

Some heuristics on the modified I-MeDeSA are applicable to individual alerts (alert-level) and others refer to the MTM vendor system as a whole (vendor system-level) (Additional file 1.) For vendor system-level heuristics on the modified I-MeDeSA, analysts scored each heuristic one time for each vendor by considering the body of all alerts evaluated for the vendor. Scores on vendor system-level heuristics were then used in computing scores for all alerts from that vendor.

Analysts completed all scoring in REDCap [35]. For each MTM alert, analysts’ individual ratings were summed and a mean score on the modified I-MeDeSA computed. For each heuristic, we also computed the percent of analyst ratings indicating alignment with the heuristic. We did this for all alerts evaluated to produce an “overall” summary of analysts’ ratings for a given heuristic, and we also computed this separately for each alert category to determine whether specific heuristic alignment differed by alert category. All computations were performed using SPSS (IBM, v. 24, Armonk, NY) [36]. Focus was made on heuristics where ≤50% of analysts’ ratings indicated alerts aligned with the heuristic. Analysts’ explanatory comments were also considered when summarizing findings and recommendations for future alert design for MTM. In an effort to provide comprehensive reporting of this evaluation, we consulted the STAR-HI statement in the preparation of this paper [37]. This evaluation was approved by the Purdue University Institutional Review Board (IRB; study number 1608018057.) Pharmacists provided written informed consent; patient consent was waived by the IRB.

## Results

From April 2017 to March 2018, nine pharmacists, representing eight pharmacies, submitted data for a total of 77 MTM alerts and we selected a purposeful sample of 24 MTM alerts for inclusion in our heuristic evaluation (Table 1.) Screenshots are not provided here given the commercial, proprietary nature of MTM vendor systems.

**Table 1:** Summary of MTM alert screenshots evaluated

Alert Category	N^a^	MTPs Targeted by Alert (number of alerts evaluated)
Indication	5	Need for ACE/ARB^b^ therapy (2)
		Need for statin therapy (1)
		Duplicate/unnecessary beta blocker drug therapy (2)
Effectiveness	5	Drug-drug interaction to reduce plasma concentration of immunosuppressant drug (1)
		Sub-optimal statin dosage (1)
		Sub-optimal choice of cholesterol-lowering drug (2)
		Sub-optimal asthma drug (1)
Safety	6	Unsafe drug (anti-hypertensives; benzodiazepine; hypnotic; antidepressant) for patient due to patient age (5)
		Drug-drug (anti-hypertensives) interaction (1)
Adherence	6	Medication (sleep agent; antidepressants, cholesterol-lowering drug, anti-hypertensive, anti-diabetic) non-adherence (6)
Cost	2	Cost-savings opportunity through switch to alternative drug (statin; anti-hypertensive) (2)

^a^ n = number of alerts evaluated for each category. (Total *N* = 24)

^b^*ACE* angiotensin-converting enzyme inhibitor, *ARB* angiotensin II receptor blocker

### Overall findings across MTM alert categories

Overall, alert categories were rated similarly to one another (Table 2.) Heuristics pertaining to *visibility* and *color* were generally met. The primary opportunities for improvement were found for heuristics related to five human factors principles (Table 3; *prioritization*; *text-based information*; *alarm philosophy*; *proximity of task components being displayed*; *corrective actions*) resulting in recommendations applicable to all, or most, MTM alert categories. One *placement* heuristic (Table 3) was noted as an improvement opportunity for many alert categories. Some heuristics related to *placement* and *corrective actions* could not be consistently assessed from MTM alert screenshots and, therefore, potential areas for improvement could not be fully elucidated.

**Table 2:** Modified I-MeDeSA scores by alert category

Score by Alert Category^a^ Mean ± SD; (Total *N* = 24)					
Indication (*n* = 5)^b^	Effectiveness (*n* = 5)	Safety (*n* = 6)	Adherence (*n* = 6)	Cost (*n* = 2)	Overall (*N* = 24)
36.2 ± 4.8	35.7 ± 5.4	37.2 ± 6.4	39.3 ± 6.6	37.8 ± 5.3	37.3 ± 5.9

^a^ Possible range of scores from 0 to 64 with higher scores indicating greater alignment with human factors heuristics, as rated by analysts

^b^each “n” refers to the number of alerts evaluated per category. Screenshots of each alert were independently rated by each of the four analysts

**Table 3:** Summary of main findings for each of the eight overarching human factors principles assessed by modified I-MeDeSA [21, 31, 32]

Human Factors Principle (number of associated heuristics on modified I-MeDeSA)	Main Findings ^a^
Principles generally met	
Visibility (3)	Across alert categories, all visibility heuristics (3i,3ii,3iii) were consistently assessed affirmatively (i.e., alerts were rated as distinguishable from the background, having appropriate color contrast, and font.)
Color (4)	All heuristics (5i-5iv), except the use of color coding (5i) to indicate specific MTP categories (e.g., minimal use of colors with clear meanings for each color), were consistently assessed affirmatively.
Principles with Improvement opportunities and/or unable to be assessed	
Alarm philosophy (1)	MTM vendor platforms did not appear to consistently have a catalog of MTPs indicating associated alerts’ priority level and expected consequences if not followed (1i.)
Prioritization (5)	Across alert categories, colors, shapes, icons, and signal words were sometimes used, but these did not clearly indicate priority (4i-4iv.) For most alert categories, color, when used, was not a redundant cue (4ii.) For patients with multiple alerts, the order of alerts was found to not clearly indicate priority (4v.)
Text-based information (10)	Heuristics pertaining to the inclusion of text to explain why the alert was shown (6ii) and the appropriateness of language for the end user (6vii) were consistently rated affirmatively. Across all alert categories, however, signal words, if used, were rated as insufficient for indicating priority (6ia.) The need for a clear consequence statement (6iv) and minimization of text (6v) were noted as enhancement opportunities for most alert categories.
Proximity of task components being displayed (1)	For most alert categories, alerts did not consistently include the information needed to support decision-making within or in close proximity to the alert. (7i)
Corrective actions (4)	Alerts did not consistently include “intelligent” corrective actions (8ia.) Alert examples could not be consistently assessed on whether the systems monitored and alerted the user to follow through with corrective actions (8ii.) For most alert categories, improvements would be needed to help prevent usability-related errors (8iii.)
Placement (4)	For many alert categories, the layout of the alert was rated as insufficient for facilitating quick information uptake by the user (2iv.) For most alert categories, alert examples could not be consistently assessed on whether the alerts appeared at appropriate times (2iii.)

^a^Roman numerals refer to specific heuristics on the modified I-MeDeSA (Additional file 1)

### Findings for specific alert categories

#### Medication adherence

Adherence alerts were assessed the most favorably overall on the modified I-MeDeSA (Table 2) and adherence was the only alert category where five specific heuristics (Table 3; Additional file 1) were favorably assessed; these pertained to the following: color as a redundant cue for alert *prioritization*, *proximity of information components* on the alert, *placement*, where an alert is linked with the medication of concern by appropriate timing, minimizing *text-based information*, and *corrective actions* to prevent usability-related errors. No improvement opportunities unique to adherence alerts were identified.

#### Medication indication and safety

Analysts’ assessments of heuristics for alerts targeting medication 1) indication and 2) safety followed the same pattern as each other, and that of alerts overall, with two exceptions. First, for indication alerts, our finding suggests that the *text-based information* provided in existing designs may place unnecessary memory load on the end-user. Specifically, existing designs require pharmacists to memorize information from other parts of the MTM vendor platform to respond to the alerts. For example, when alerts advised the pharmacist to recommend the addition of a new medication, the pharmacist would need to navigate to another screen to review pertinent information such as other medications and diagnoses in order to decide whether the recommendation was appropriate. Second, also with regards to *text-based information*, safety alerts were rated more favorably than other alerts with regards to having a clear consequence statement. Indication and safety alerts also scored the most favorably on the heuristic pertaining to *placement* of information to facilitate quick uptake.

#### Medication effectiveness and cost

Analysts’ assessment of heuristics for alerts targeting 1) effectiveness and 2) cost MTPs followed the same pattern as each other, and that of alerts overall, with no improvement opportunities unique to either category.

## Discussion

This study extends prior research on alert design to identify leverage points for MTM alerts used by community pharmacists during medication reviews. Our research addresses a growing need by focusing on pharmacists as end users, the community pharmacy setting, and MTM alerts targeting complex, multimorbid patients--for which there is an overall paucity of literature on alerts [1, 7, 38]. Although extensive human factors studies of alert design have been conducted in other domains [17, 21], recent literature reviews have noted a critical need for further attention given to human factors principles in alert design in the healthcare domain [39, 40]. This research helps address this need and we anticipate that our findings can be used by MTM vendors that serve community pharmacies across the U.S., as well as for similar pharmacist-provided medication review services in other countries, such as those in the Netherlands and Australia [41, 42], to develop enhancements to MTM alerts to encourage alignment with human factors principles. To our knowledge, this study is also the first to apply the I-MeDeSA beyond the original work focused on drug-drug interaction alerts [21, 31, 32].

### Recommendations across all MTM alert categories

We found that heuristics pertaining to *visibility* and the use of *color* were generally met by MTM alerts. We also identified opportunities for improvement that were consistent across all alert categories evaluated, as well as opportunities to strengthen specific alert categories. Our findings point to several recommendations for MTM alert design that could be considered by MTM vendors and others developing similar alerts for community pharmacists. Across all alert categories, four recommendations could be considered. First, vendors should ensure that a general catalog of MTPs, indicating associated MTM alerts priority level and severity of consequences is transparent to users to facilitate clear understanding of the MTM platform design and capabilities [43]. This could be an electronic catalog, where these underlying components of the platform are conveyed to pharmacists via the alerts themselves, a help guide, via a dashboard, or another mechanism. Systematized nomenclature of medicine-clinical terms (SNOMED CT) could be used to guide this process [44]. This improvement opportunity aligns with results of Marcilly et al.’s 2015 review of usability flaws of medication alerts which identified “function is not transparent enough to the user,” as a flaw, specifically noting a lack of transparency for “how alerts are categorized by severity.” [45] Second, clear alert definitions and consistent use of colors, shapes, icons, and signal words to indicate clinical priority may improve usability and better support decision making. Third, MTM alerts should display in order of clinical priority on the screen; for example, serious safety concerns could be displayed first. Fourth, to better support pharmacists in taking appropriate corrective actions, MTM alert design should facilitate direct communication between pharmacists and prescribers and integrate directly with pharmacy dispensing systems regarding pharmacist recommendations (e.g., to hold a prescription from being filled, pending prescriber approval of a dose change suggested during the CMR.) In summary, these four recommendations could be used to strengthen the design of MTM alerts, across all categories evaluated.

### Recommendations for most MTM alert categories

Except for adherence alerts, which were stronger overall, we propose four additional recommendations for alerts based on our findings. First, signal words, such as “major drug-drug interaction,” are needed to denote clinical priority and should be used with other features such as colors, shapes, and icons as redundant visual cues. Color was the primary cue for many alerts in our study, but redundancies are needed to support users with color deficiencies. Similarly, a recent review of medication alerts as part of computerized prescriber order entry identified the need for better worded alerts utilizing explicit language [40]. Second, alerts should be designed to enable the user to access relevant information (e.g., by clicking an “info button”) directly from the alert. This aligns with the findings of three recent literature reviews which highlight the potential benefits of embedded laboratory data as well as “info buttons” to provide relevant information to users as part of medication alerts [40, 45, 46]. Third, based on warnings literature [33] alert language should be concise, displaying short statements or bullet points rather than complete sentences. Consistent with our findings, the use of lengthy text was identified as a common problem during another recent heuristic evaluation of physician consultation order templates [26]. Fourth, changes could be made to prevent potential usability-related errors. This includes changes to reduce extraneous clicks required from the pharmacist to improve efficiency. For instance, design changes could be made to reduce the potential for “pick list” errors [47] by removing some of the drop-down menus and replacing them with radio buttons or checklists.

### Recommendations for specific MTM alert categories

In addition, all alerts should include a consequence statement that clearly informs the user as to what might happen if the alert were ignored. This was identified as an improvement opportunity for most alert categories, with safety alerts being the exception because they consistently included a consequence statement already. For example, a consequence statement for an indication-related MTM alert of “needs additional drug therapy” could read, “Lack of statin therapy for a patient with a high risk for cardiovascular disease will increase their likelihood for a heart attack or stroke.” Moreover, with the exception of indication and safety alerts, alert design could be improved to facilitate quick uptake of information by the end user by moving critical information to the top and left position in the alert screen to align with the reading process. Spacing should also be used to separate the most critical information from other details, critical information should be grouped, and bullet points should be used in place of prose [33]. Finally, for indication alerts, changes are recommended to minimize the memory load of the end user by providing key patient-specific information on the alert that is needed for decision-making; examples include a summary of medications the patient tried in the past along with relevant laboratory data.

### Comparison with findings for EHR alerts

For some heuristics, our findings align with Phansalkar et al.’s results for EHR drug-drug interaction alerts [32]. For example, *visibility* heuristics were rated favorably in both their evaluation and ours as well. Interestingly, this contrasts with the findings of a heuristic evaluation of an electronic medication administration record where violations of visibility heuristics were common [48]. In addition, both ours and Phansalkar’s evaluations found improvement opportunities for *alarm philosophy* and *prioritization* heuristics [32]. In contrast, whereas *proximity of task components* was assessed favorably for EHR drug-drug interaction alerts [32], we found improvement opportunities for this heuristic for most categories of MTM alerts. Moreover, while the *placement* of information was assessed favorably for EHR alerts [32], we found improvement opportunities for some alert categories with regards to the alert layout needing to better facilitate quick uptake of information.

### Limitations

There are limitations to our evaluation. First, for most alert categories, we were unable to consistently assess whether alerts were linked with the medication by appropriate timing. This is due to the static nature of the screenshots used in our evaluation; in contrast, the reviewers who evaluated the EHR alerts for Phansalkar et al. requested a walk-through (via web-conference) of the EHR vendor systems when a screenshot was insufficient for evaluating a heuristic [32]. Second, in spite of our efforts to identify alert examples for each category from both MTM vendors, cost alerts were obtained from only one MTM platform. Moreover, it is important to note that different types of specific MTPs are represented within each category. While we made an effort to sample alerts targeting a range of specific MTPs for each category (Table 1), we did not require the same number of each MTP per category nor did we capture the same specific alerts from each vendor. Our study is unlikely to capture all variation of alert design within a given alert category. Therefore, our recommendations might vary in applicability to specific alerts within each category/vendor. Future research could focus on a specific alert category and further assess effective strategies to improve the design of alerts for specific MTM alert categories. Third, while pharmacist investigators applied a specific well-accepted taxonomy for MTPs to categorize alerts [23], different pharmacists or the application of a different taxonomy for MTPs might have resulted in different categorization. Fourth, we believe that at least one of the MTM vendors made some changes to alerts during our data collection period, which could have influenced our findings. Fifth, while some commonalities across the EHR drug-drug interaction alert evaluation [32] and our current MTM alert evaluation were noted, direct comparisons of I-MeDeSA scores from the two evaluations are not possible since we modified the I-MeDeSA language, added heuristics, and modified the scoring approach. Moreover, it is important to note that an overall numeric score on the modified I-MeDeSA alone does not provide insight into the severity of heuristic violations and any changes in I-MeDeSA scores following changes in alert design could reflect a particular heuristic being met while violating other heuristics. In any heuristic evaluation, quantitative scores should serve as a guide to note potential violations and should be interpreted in the context of qualitative data. Finally, our evaluation only assessed MTM alerts for alignment with heuristics included in the modified I-MeDeSA. Other recommendations for medication alert design exist, including those described by Pelayo et al. [49]

### Opportunities for future research

Our findings point to several opportunities for further research. For example, there is a need for formal usability testing with community pharmacists to identify potential usability errors that might not be detected by a heuristic evaluation of screenshots. Moreover, heuristic evaluation relies on experts’ analyses, but as emphasized in recent literature reviews, user-centered design principles are paramount in alert design [39, 40]. Therefore, data collected directly from end users (i.e., pharmacists) should also be considered in MTM alert re-design and future research should examine the perspectives of pharmacists and other end users involved in resolving MTM alerts. Studies are also needed to evaluate any alert enhancements made in response to recommendations informed by this evaluation. A prior simulation study where usability testing was conducted with EHR alerts and prescribers demonstrated that redesigning EHR alerts to better align with human factors principles increased prescribers’ efficiency and reduced prescribing errors, however it is unknown whether MTM alert changes would result in similar positive outcomes [50].

## Conclusions

This is one of the first studies to evaluate MTM alerts and the first, to our knowledge, to evaluate MTM alerts used by community pharmacists for alignment with human factors principles. We found that MTM alerts generally have opportunities for improvement related to several human factors principles, especially as related to alert *prioritization*, *text-based information*, *alarm philosophy*, and *corrective actions*. We expect that our findings and recommendations can be used by MTM vendors and community pharmacies across the U.S. to develop enhancements to MTM alerts. The subsequent changes made to MTM alerts are expected to enhance usability for pharmacists and improve patient outcomes. MTM alert recommendations have not yet been implemented and tested, however, and should be evaluated in future research.

## Additional file

Additional file 1: Modified I-MeDeSA. Contains a copy of the modified I-MeDeSA used to guide this heuristic evaluation. (DOCX 23 kb)

## Data Availability

Data sharing is not appropriate for this article as this heuristic evaluation reports on a very small sample size of alert screenshots.

## Abbreviations

ADEs: Adverse drug events
CMR: Comprehensive medication review
EHR: Electronic health record
I-MeDeSA: Instrument for Evaluating Human-Factors Principles in Medication-Related Decision Support Alerts
IRB: Institutional Review Board
MPPBRN: Minnesota Pharmacy Practice-based Research Network
MTM: Medication therapy management
MTP: Medication therapy problem
Rx-SafeNet: Medication Safety Research Network of Indiana
SEIPS: Systems Engineering Initiative for Patient Safety
SNOMED-CT: Systematized nomenclature of medicine-clinical terms
STAR-HI: Statement on reporting of evaluation studies in health informatics
