# Supplementary material for: Alerts for community pharmacist-provided medication therapy management: recommendations from a heuristic evaluation
Source: BMC Med Inform Decis Mak. 2019 Jul 16;19:135. doi: 10.1186/s12911-019-0866-0 (PMC6636156; doi:10.1186/s12911-019-0866-0)
Supplement: Supplementary file 1 — Modified I-MeDeSA. Contains a copy of the modified I-MeDeSA used to guide this heuristic evaluation. (DOCX 23 kb) [file 12911_2019_866_MOESM1_ESM.docx]

Additional File 1. Modified I-MeDeSA^a,b^

| Alarm philosophy | |
| --- | --- |
| 1i | *[****Vendor****] Does the system provide a general catalog of medication-related problems, correlating the priority level of the alert with the severity of the consequences?* |
| Placement | |
| 2i | *[****Vendor****] Are different types of alerts meaningfully grouped? (i.e., by the severity of the alert, where all level 1 alerts are placed together, or by medication, where alerts related to a specific medication are grouped together)* |
| 2ii | Is the response to the alert (e.g., way to document actions taken to resolve problem) provided along with the alert, as opposed to being located in a different window or in a different area on the screen (e.g., external to the alert)? |
| 2iii | Is the alert linked with the medication by appropriate timing? (i.e., a drug-drug interaction alert appears as soon as a drug is chosen/entered and does not wait for the user to complete data entry and then alert him/her about a possible interaction) |
| 2iv | Does the layout of critical information contained within the alert facilitate quick uptake by the user? Critical information (see Text Based information below) should be placed on the first line of the alert or closest to the left side of the alert box. The most important information should appear in larger font and/or at the top of the alert. |
| Visibility | |
| 3i | Is the area where the alert is located distinguishable from the rest of the screen? This might be achieved through the use of a different background color, a border color, highlighting, bold characters, occupying the majority of the screen, etc. |
| 3ii | Is the background contrast sufficient to allow the user to easily read the alert message? (i.e., dark text on a light background is easier to read than light text on a dark background) |
| 3iii | Is the font used to display the textual message appropriate for the user to read the alert easily? (i.e., a mixture of upper and lower case lettering is easier to read than upper case only) |
| Prioritization | |
| 4i | *[****Vendor****] Is the prioritization of alerts indicated appropriately by color? (i.e., colors such as red and orange imply a high priority compared to colors such as green, blue, and white.* |
| 4ii | If color is used, is it used as a redundant cue? Due to color blindness, color should NOT be the only indicator of alert priority.) |
| 4iii | *[****Vendor****] Are signal words* *consistently and appropriately assigned to each existing level of alert to indicate clinical priority? (e.g., ‘note,’ ‘warning,’ or ‘danger’, ‘high risk’ or other terms) [A signal word is 1-3 words or so to capture attention]* |
| 4iv | Does the alert utilize shapes or icons in order to indicate the priority of the alert? (i.e., angular and unstable shapes such as inverted triangles indicate higher levels of priority than regular shapes such as circles) |
| 4v | *[****Vendor****] In the case of multiple alerts for the same patient, are the alerts placed on the screen in the order of their importance? The highest priority alerts should be visible to the user without having to scroll through the window.* |
| Color | |
| 5i | *[****Vendor****] Does the alert utilize color-coding to indicate the type of medication-related problem? (i.e., drug–drug interaction vs. allergy alert vs. possible medication non-adherence)* |
| 5ii  5iii  5iv | Is color minimally used to focus the attention of the user? As excessive coloring used on the screen can create noise and distract the user, there should be less than 4 colors used in a given alert.  *[****Vendor****] There should be no more than 7 colors total used by the vendor for alerts.^b^*  Is the meaning of each color adequately conveyed?^b^ |
| Text-based information: Does the alert possess the following information components? | |
| 6i  6ia | A signal word is present on the alert (e.g., ‘note,’ ‘warning,’ or ‘danger’, ‘high risk’ or other terms) [1 to 3 words to capture attention]  If yes, does the signal word or text shown near/with signal word indicate clinical priority?^b^ |
| 6ii | A statement of the nature of the medication-related problem describing why the alert is shown. This may be a generic statement in which, for example, interacting classes are listed, or an explicit explanation in which specific drug-drug interactions are clearly indicated. |
| 6iia | If yes, are the specific medication-related problem(s) (e.g., interacting drugs, possible medication non-adherence) explicitly indicated to explain why the alert is appearing? |
| 6iii | An instruction statement (telling the user about the desired action or how to avoid the undesired clinical/patient outcome.) |
| 6iiia | If yes, does the order of recommended tasks reflect the order of required actions? |
| 6iv | A consequence statement telling the user what might happen if the alert is ignored; (medical or patient care consequences, for example, the allergic reaction that may occur if the instruction information is ignored.) |
| 6v | Does the alert minimize the use of text? (e.g., short statements rather than long complex statements or complete sentences) |
| 6vi. | Does the alert minimize the memory load of the end-user? (Users should not have to memorize a lot of information from other parts of the system in order to respond to the alert. e.g., an alert for medication non-adherence should present prescription fill data, etc.)^b^ |
| 6vii. | Is all of the language in the alert likely to be understandable to the intended end-users (pharmacists)? (e.g., avoids use of abbreviations, unfamiliar technical terms)^b^ |
| Proximity of task components being displayed | |
| 7i | Are the informational components needed for decision making on the alert present either within or in close spatial and temporal proximity to the alert? For example, is the user able to access relevant information directly from the alert, that is, a drug monograph, an ‘infobutton,’ or a link to a medical reference website providing additional information? |
| Corrective actions | |
| 8i | Does the system allow corrective actions that also serve as an acknowledgement of having seen the alert? (A corrective action is an intervention or response that the alert is not relevant.) |
| 8ia | If yes, does the alert utilize intelligent corrective actions that allow the user to complete a task? For example, if warfarin and ketoconazole are co-prescribed, the alert may ask the user to ‘Recommend warfarin dose be reduced by 33–50% and follow the patient closely.’ An intelligent corrective action would be ‘Monitor patient AND recommend warfarin dose be reduced by 33–50%.’ Selecting this option would simultaneously over-ride the alert AND direct the user back to a screen where the user can prepare prescriber recommendations. |
| 8ii | *[****Vendor****] Is the system able to monitor and alert the user to follow through with corrective actions? Referring to the previous example, if the user tells the system that he/she will recommend a reduced warfarin dose but fails to follow through on that promise, does the system alert the user?* |
| 8iii | Does the alert design help prevent usability-related errors? (e.g., extra clicks, easy to enter wrong information; sources of confusion; too much scrolling, inappropriate use of ‘check all’ vs. ‘check one’; inappropriate use of defaults. etc.)^b^ |

a: Heuristics in italics are at the vendor level

b: Heuristic was added by research team
